# Real-Time TEM Observation of the Role of Defects on Nickel Silicide Propagation in Silicon Nanowires

**DOI:** 10.1021/acsnano.4c01060

**Published:** 2024-03-21

**Authors:** Temilade Esther Adegoke, Raman Bekarevich, Hugh Geaney, Sergey Belochapkine, Ursel Bangert, Kevin M. Ryan

**Affiliations:** † Department of Chemical Sciences and Bernal Institute, University of Limerick, Limerick, V94 T9PX Ireland; ‡ Advanced Microscopy Laboratory, Centre for Research on Adaptive Nanostructures and Nanodevices (CRANN), Trinity College Dublin, Dublin, D02 DA31 Ireland; § Bernal Institute, University of Limerick, Limerick, V94 T9PX Ireland; ⊥ Department of Physics and Bernal Institute, University of Limerick, Limerick, V94 T9PX Ireland

**Keywords:** *in situ* transmission electron microscopy, phase transitions, defects, nickel silicide, nanowires

## Abstract

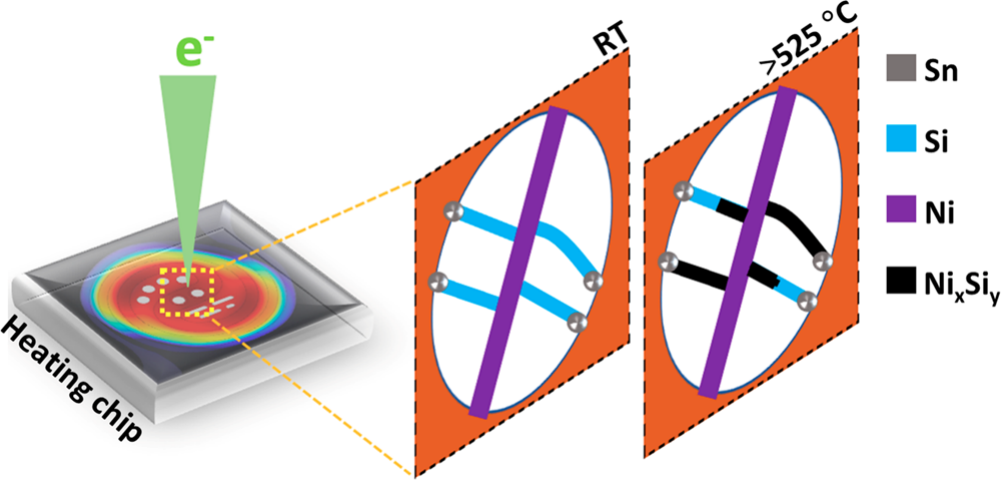

Metal silicides have received significant attention due to their high process compatibility, low resistivity, and structural stability. In nanowire (NW) form, they have been widely prepared using metal diffusion into preformed Si NWs, enabling compositionally controlled high-quality metal silicide nanostructures. However, unlocking the full potential of metal silicide NWs for next-generation nanodevices requires an increased level of mechanistic understanding of this diffusion-driven transformation. Herein, using *in situ* transmission electron microscopy (TEM), we investigated the defect-controlled silicide formation dynamics in one-dimensional NWs. A solution-based synthetic route was developed to form Si NWs anchored to Ni NW stems as an optimal platform for *in situ* TEM studies of metal silicide formation. Multiple *in situ* annealing experiments led to Ni diffusion from the Ni NW stem into the Si NW, forming a nickel silicide. We observed the dynamics of Ni propagation in straight and kinked Si NWs, with some regions of the NWs acting as Ni sinks. In NWs with high defect distribution, we obtained direct evidence of nonuniform Ni diffusion and silicide retardation. The findings of this study provide insights into metal diffusion and silicide formation in complex NW structures, which are crucial from fundamental and application perspectives.

Silicon nanowires (Si NWs) have attracted significant research interest as functional nanomaterials for various applications including nanoelectronics,^1,2^ optoelectronics,^3^ sensors,^4^ and energy storage.^5,6^ In particular, silicides, which are compounds of metals with Si, have been widely investigated as metal contacts for Si NW device applications due to their excellent compatibility with Si. Among the various silicide NWs formed, Ni silicide NWs offer the lowest resistivities, making them suitable as interconnects.^7−9^

Unsurprisingly, different synthetic approaches have been developed for the formation of Ni silicide NWs. The most typical synthetic approaches include silane (SiH_4_) delivery to Ni substrates via chemical vapor deposition (CVD)^10−13^ and solid-state reactions^14^ involving the annealing of presynthesized Si NWs coated with Ni.^7,15−17^ Both approaches have allowed the synthesis of different Ni silicide phases from metal-rich to Si-rich. However, an advantage of solid-state reaction over CVD is that axial heterostructures consisting of Ni silicide and Si segments can be formed by incorporating lithographic processes to selectively pattern localized regions of the Si NW.^7^ Ni silicide/Si NW heterostructures are particularly of interest in nanoelectronics, for example, in single-NW transistor devices. In this configuration, the end of the Si NW contacts Ni pads, providing electrical conductivity to the Si NW.^14,17^

Sheehan et al. demonstrated the possibilities of synthesizing axial heterostructure NWs consisting of Ni silicides and Si segments from a Ni substrate using a high boiling point solvent-based system.^18,19^ They initially formed Au-seeded Si NWs, and in the same reaction Ni diffusion from the substrate to the Si NW led to Ni silicide formation via solid-state diffusion. In this synthesis approach, varying degrees of silicidation were achievable, ranging from Ni silicide/Si NW heterostructure NWs to full Ni silicide NWs. This variation was attributed to the defect distribution in the NWs during the initial stages of Si NW growth following postmortem characterization. However, a comprehensive understanding of this silicidation process necessitates real-time studies of solid-state reactions at the nanoscale, achievable through *in situ* transmission electron microscopy (TEM) techniques. *In situ* TEM heating experiments can reveal transient states, leading to a complete mechanistic understanding of complex chemical processes.

In this work, *in situ* TEM is used to track Ni silicide formation. The NWs in question required the development of a bespoke synthetic approach, with Sn-catalyzed Si NWs directly grown from Ni NW stems via a solution-based route. The Ni stem provides an excess Ni supply for silicide formation without any need for a lithography process.^18^ NWs with varying morphologies and crystalline defects were selected to determine the reaction kinetics in Ni silicide systems. In addition, insights into silicide propagation in the presence of surface impurities and Sn dopants from the catalyst are presented. The approach allows for the correlation between Ni silicide growth rate and defect distribution to be unravelled.

## Results and Discussion

**Figure 1:**
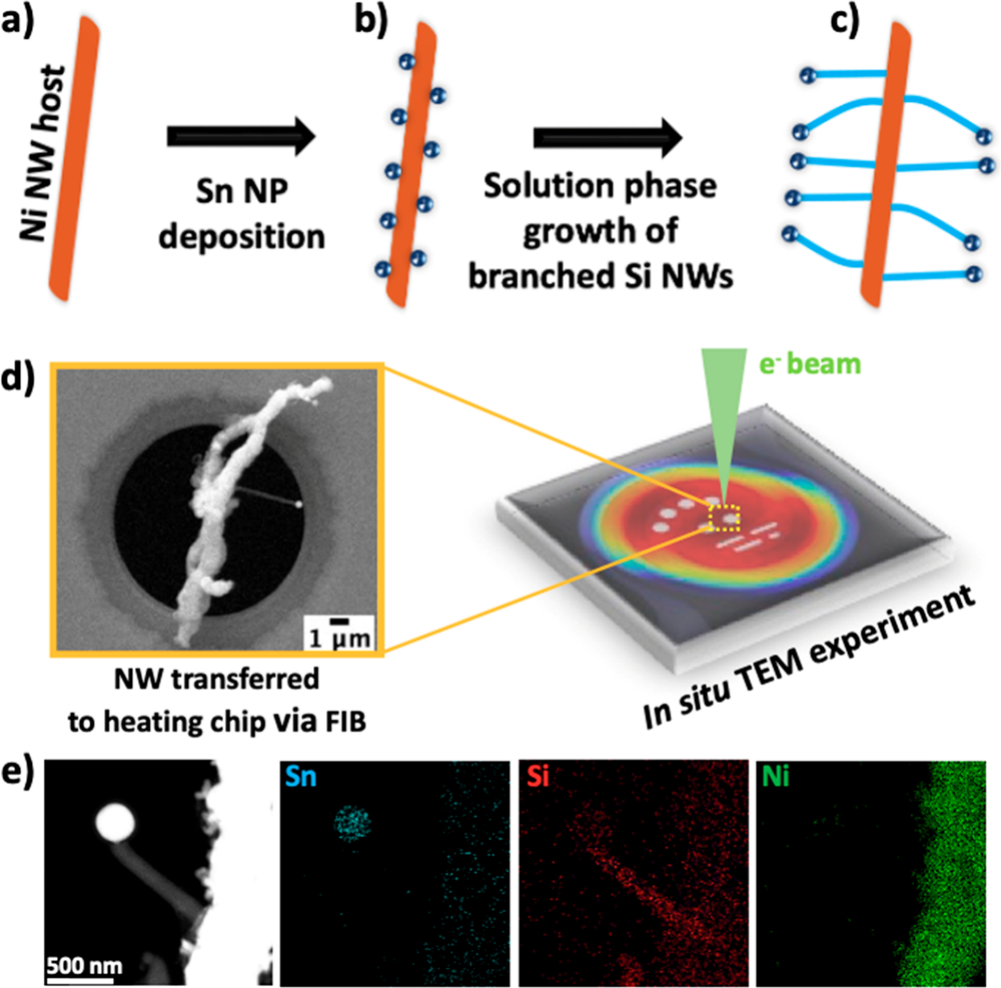
Overview of sample preparation for *in situ* heating experiment. (a–c) SLS growth of Si–Ni branched NWs through the deposition of Sn nanoparticles (NPs) on the sidewall of the Ni NW stem and the introduction of liquid silicon precursor. (d) Top view of NW transferred to a heating chip for *in situ* TEM heating experiment. (e) HAADF STEM image of Si–Ni branched NW with elemental maps of Sn (blue), Si (red), and Ni (green) regions of the NW.

Figure 1 shows an overview of the sample preparation methods, eliminating the lithographic processes required for such solid-state reactions. First, Sn-seeded Si NWs were grown from the sidewalls of a Ni NW stem, which also served as the Ni reservoir (Figure 1a–c). The as-grown Si–Ni branched NWs were transferred to the viewing area of the heating chip via a FIB pick-and-place technique in preparation for *in situ* TEM experiments (Figure 1d).

HAADF-STEM images of the as-grown Si–Ni branched NWs with corresponding elemental composition analysis are shown in Figure 1e. The differences in atomic number (*Z*) between Sn, Si, and Ni provide sufficient contrast in the HAADF-STEM image, highlighting the contrast difference between the Si segment and the Sn/Ni segments. STEM energy dispersive X-ray spectroscopy (EDX) elemental maps confirmed the localization of Ni to the stem with minor traces of Sn along the Si NW segment. To investigate the diffusion behavior of Ni in Si NWs and the different silicide phases formed, two types of Si–Ni branched NWs were studied: (i) straight Si NWs and (ii) kinked Si NWs. In addition, data on the dynamic behavior of the Sn catalyst in response to the energy from the electron beam and the high temperatures required for silicidation of the Si NWs are presented.

### Straight Si NWs

**Figure 2:**
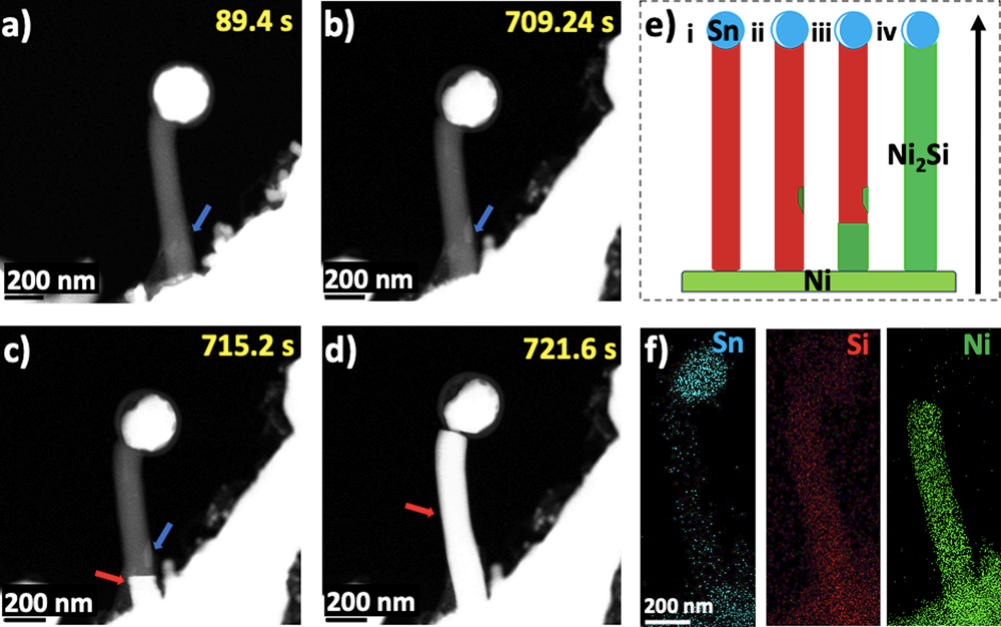
Nickel silicide formation in a Si NW with a ⟨111⟩ growth direction. (a–d) HAADF-STEM snapshots recorded during Ni diffusion at 900 °C. (e) Schematic diagram of the stages of silicide growth. (f) HAADF-STEM image and corresponding EDX compositional maps of Sn (blue), Si (red), and Ni (green).

Figure 2 shows the structural evolution of a ⟨111⟩-oriented Si NW (*d* = 161 nm, *l* = 812 nm) annealed at 900 °C for 12 min (Figure S1a, Movie S1). Ni initially diffuses through a surface impurity (Figure S2a, Figure 2a) at the edge of the Si NW (blue arrow). STEM EDX compositional analysis showed that the impurity region contained a high percentage of carbon, suggesting that the impurity in the NW originated during the FIB lift-out techniques used for site-specific sample preparation (Figure S2b). The impurity seems to be the preferred nucleation site and acts as a Ni sink, channelling more Ni atoms diffusing from the Ni source. As Ni accumulates, it forms a discrete layer along the Si NW, highlighted by the blue arrow in Figure 2a–c. At the interface between the Ni stem and the Si NW, Ni diffuses and reacts with Si to form a Ni_*x*_Si_*y*_/Si heterostructure NW (red arrow), which consumes 167 nm of the length of the Si NW after 715.2 s of annealing. After the heterostructure formation, there is a jump in Ni propagation, consuming the remaining 645 nm length of the Si NW (Figure 2d), which led to complete silicidation of the NW (Figure 2e). The sudden jump in Ni diffusion within a 6.4 s window can be attributed to the high annealing temperature. This observation was consistent with other NW samples annealed above 700 °C (Figure S3). In general, Ni silicide formation in Si nanowires is considered to occur either by surface diffusion or volume diffusion of Ni atoms.^14,20^ Depending on the NW surface-to-volume ratio, surface diffusion could dominate with decreasing Si NW diameter.^21,22^ The observed increase in the silicide propagation speed in these nanowires (Figures 2 and S3) may be attributed to surface diffusion, considering the combined effects of the nanoscale size effect and high temperature.

The contrast change in the NW during annealing indicated the formation of a silicide. However, to further confirm the presence of Ni, EDX elemental maps were recorded after annealing (Figure 2f). Using STEM-EDX map analysis, the relative composition of this silicide NW was 73% Ni and 27% Si (Figure S4a,b), which would suggest that this intermediate phase was Ni_2_Si. HRTEM analysis of the Ni_2_Si NW (Figure S1b) viewed down the [001] zone axis revealed the presence of a lateral twin boundary (TB). Fast Fourier transform (FFT) patterns (Figure S1c,d) were extracted from either side of the boundary and indexed for orthorhombic Ni_2_Si (*Pnma*; *a* = 5.368 Å, *b* = 3.713 Å, *c* = 7.0872 Å), further confirming the formation of a Ni-rich phase. TBs in NWs have been shown to initiate irregular growth behavior, preventing silicide propagation from one side of the boundary to the other.^23^ A previous report showed that at lower temperatures (300 °C), TBs in NWs initiate irregular growth behavior, preventing silicide propagation from one side of the boundary to the other.^23^ However, in our present study (Figure 2) we do not observe the influence of the TB defect on the silicide propagation. It can be suggested that the high annealing temperature (900 °C) overcomes the energy barrier associated with the TB, resulting in uniform silicide growth.

**Figure 3:**
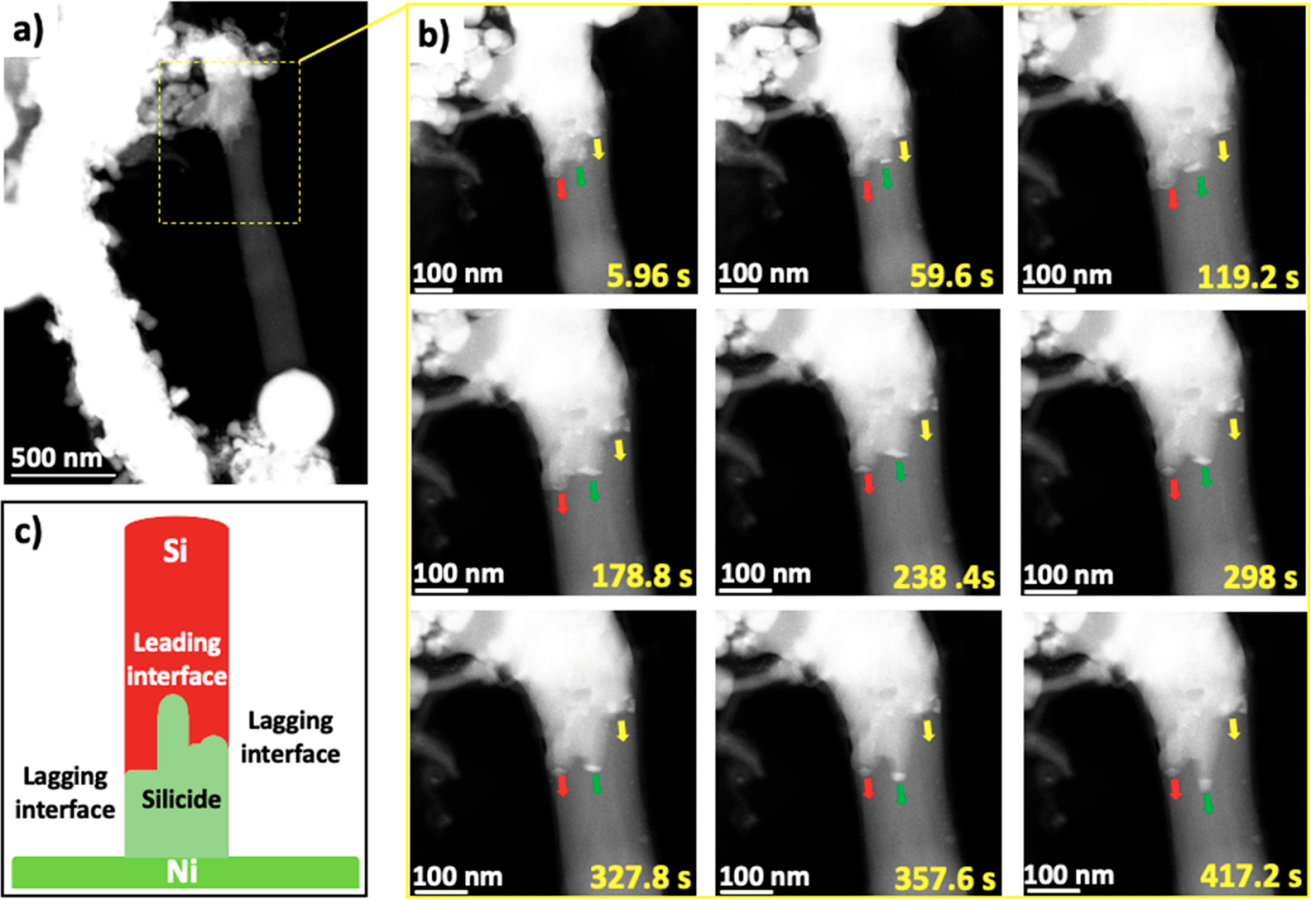
(a) Low-magnification HAADF-STEM images of ⟨111⟩ Si NW grown from a Ni NW stem. (b) Time-lapse HAADF-STEM image series following the nonuniform silicide growth at 550 °C. The colored arrows represent different silicide growth fronts. (c) Schematic illustration of silicide growth along the Si NW.

Investigations of silicide formation in a different ⟨111⟩-oriented Si NW (Figure 3a) annealed at 550 °C (Figure S5, Movie S2) showed an uneven growth behavior. Three different silicide growth fronts were observed, indicated by the colored arrows (Figure 3b). The growth fronts can be observed at the left, center, and right segments of the NW with varied silicide growth rates. The center of the NW (green arrow) becomes the leading growth front (*t* = 327.9 s) and grows rapidly while the other segments are lagging. The rapid growth rate indicates that the center of the NW is favorable for Ni diffusion and silicide nucleation. As illustrated in Figure 3c, the silicidation is driven by the diffusion of Ni into Si and then heterogeneously nucleates at different segments of the NW, forming an uneven interface between the silicide and pure Si phase (Figure S6).

**Figure 4:**
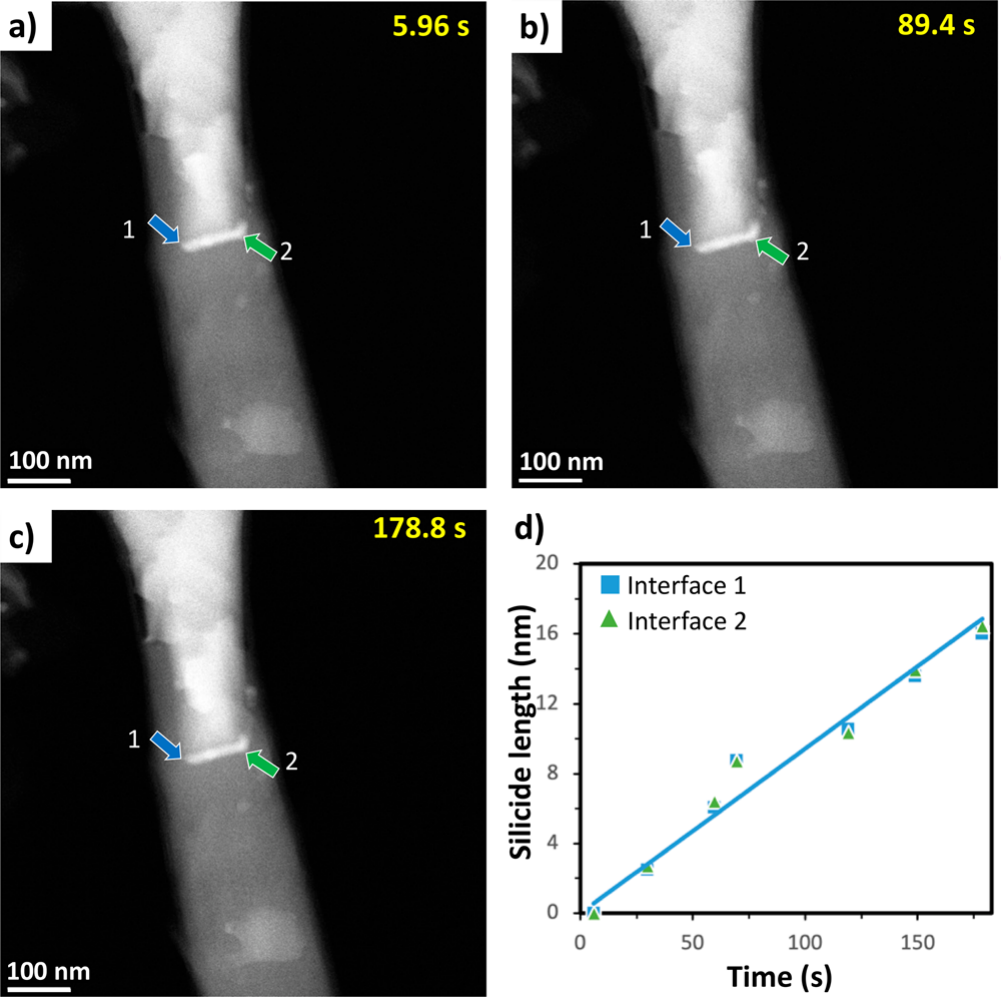
(a–c) Time-lapse HAADF-STEM image series following the leading silicide growth front at 600 °C. Two interfaces exist and are highlighted with blue and green arrows. (d) The plot of the silicide lengths for each interface vs annealing time.

Further investigation (Figure 4a–c, Movie S3) shows that the leading growth front at the center of the NW has evolved into a more uniform and linear silicide growth owing to the increase in temperature to 600 °C and annealing time of 27 min. The width of the leading growth front also increased by a factor of 4, and the growing silicide layer is controlled by the diffusion rate of the Ni atoms through the Si NW. Two parallel silicide interfaces are observed, marked with blue (interface 1) and green (interface 2) arrows in Figure 4a–c. The plot in Figure 4d shows that the silicide growth front increases linearly as a function of reaction time. Visibly, the growth rates of both interfaces are almost the same (Figure 4d). Similar to the first straight Si NW studied, Ni atoms diffuse through surface impurities in the Si NW, forming discrete regions of low Ni concentrations observed down the NW length in Figure 4a–c, appearing as a layer with a less bright contrast compared with the silicide segment.

**Figure 5:**
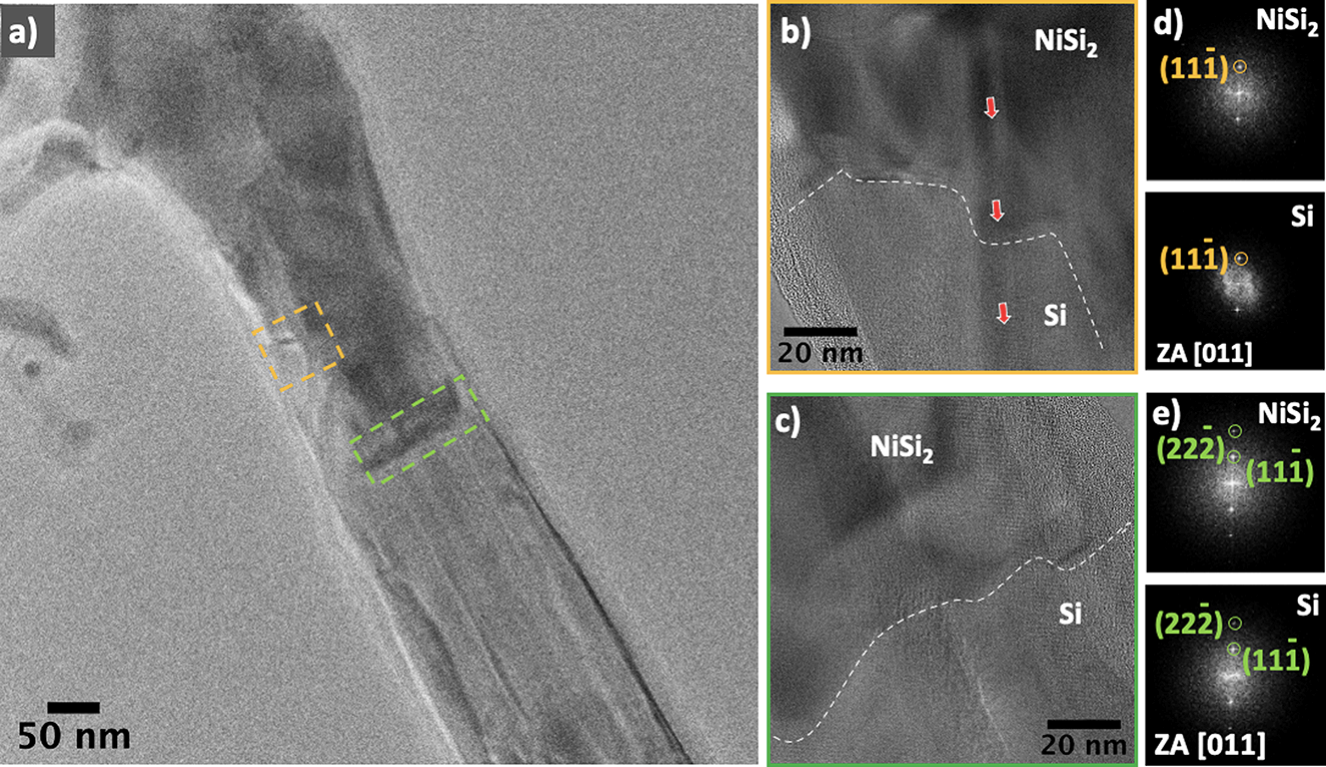
(a) Low-magnification TEM image of NW annealed at 600 °C. (b) HRTEM image of the lagging interface in the orange dashed box in panel a. (c) HRTEM image of the leading interface in the green dashed box in panel a. (d) FFT patterns of NiSi_2_ and Si segments in b. (e) FFT patterns of NiSi_2_ and Si segments in c. The white dashed lines represent the NiSi_2_/Si at the interface, and the red arrows highlight the strain in the NiSi_2_/Si NW.

As observed in Figure 4, there is still a lagging interface located behind the leading growth front. The retardation in the diffusion of Ni was linked to the high degree of strain buildup in the NW (Figure 5), creating additional energy barriers in the system. At the region where the growth rate of silicide is retarded (Figure 5b), the strain accumulated in the nanowire (red arrows) leads to uneven silicide growth. Figure 5c shows the leading silicide segment of the NW with less strain and uniformity. Interestingly, even with strain, an epitaxial interface is formed between the silicide/Si NW. FFT patterns (Figure 5d,e) extracted from the HRTEM images show the close similarity in crystal structure between the silicide and Si segments. The indexed FFT of the silicide indicated a cubic NiSi_2_ phase, indicating that the Ni supply was limited while Si was abundant.^24−26^ This Si-rich phase NiSi_2_ formed can be attributed to the contact quality between the Si NW and the Ni stem.

By analyzing the HRTEM images of the interfaces with geometric phase analysis, we measured the strain gradients in the nanowire (Figure S7a,e). Maps of the strain field components of the (ε_*yy*_, ε_*xx*_, and ε_*yx*_) are shown in Figure S7b–d and f–h. The contrast resulting from compressive and tensile strain is visible in the deformation tensor maps of ε_*xx*_ and ε_*yy*_. Notably, the values of ε_*xx*_ and ε_*yy*_ at the leading interface (Figure S7f–h) reveal areas where the strain is slightly relaxed. This demonstrates the influence of strain on the silicide growth front, and factors inducing the strain were considered. Some studies have attributed strain accumulation to compressive stress from the Si native oxide on the NW surface and volume expansion or lattice mismatch associated with phase transformation.^20,27−30^ However, strain due to volume expansion and lattice mismatch can be ruled out here due to the NiSi_2_ phase formed since NiSi_2_ and Si have the same cubic lattice structure and also have close lattice parameters, which are 0.542 and 0.543 nm, respectively.^17,24,31^ At room temperature the unit volume of Si is 20.01 Å^3^, while for NiSi_2_ the silicide volume per Si atom is 19.75 Å^3^, respectively.^20,24^

TEM images of the Si NW before annealing (Figure S8a,b) show the presence of defects that appear as contrast lines along the NW. These line imperfections within the NW are characteristics of screw dislocations, twin boundaries, or stacking faults.^32−34^ The presence of defects introduces localized strain into the lattice structure of the NW. The strain is further enhanced during the annealing experiment. The high-resolution TEM image of the Si NW after annealing (Figure S8c–f) reveals the presence of a high density of planar defects including twin boundaries and stacking faults. Planar defects require low formation energy, which is typically 28 mJ m^–2^ (22 meV/bond) to form twin defects in Si NWs and is half the energy to form stacking faults.^35−37^ These planar defects are often generated in seeded NWs grown via vapor or solution phase, which can be linked to the inherent instability of the metal seeds and growth conditions.^38−43^ Furthermore, the Si NW sample exhibited variations in diameter along its lengths (Figure S9). This nonuniform growth process of the NW may also lead to structural defects, contributing to the high distribution of planar defects in the NW.^44^ Given these factors, we can summarize from this sample that uneven silicide resulted from the structural defects in the NW. The results provide qualitative support for the origin of the irregular silicide growth behavior, which compliments *ex situ* analysis on metal diffusion.^18^

**Figure 6:**
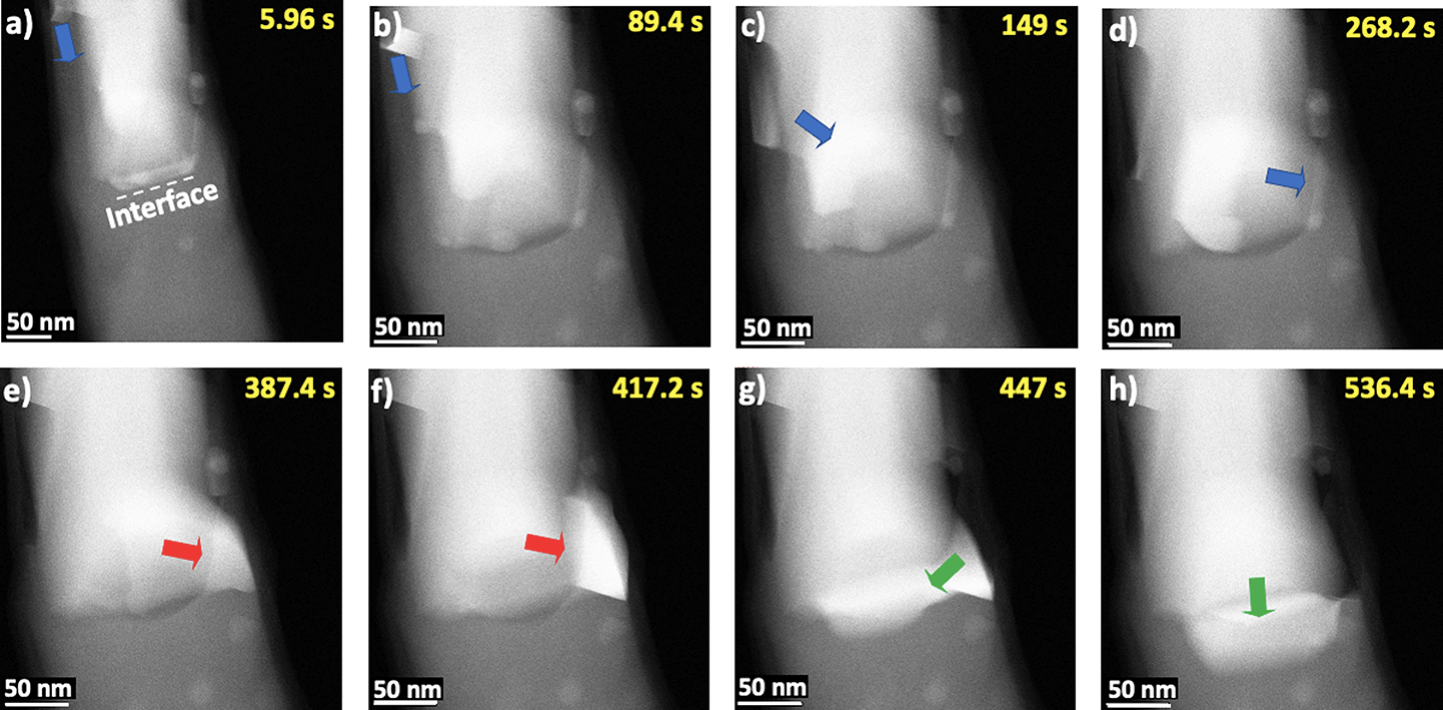
(a–h) Time-lapse HAADF-STEM image series for the sample further annealed at 625 °C. The blue arrows are used to track the path of the moving region. Red arrows highlight a diffusion pathway for the moving region, and green arrows highlight gradual interfacial segregation.

The Ni silicide growth rate can be tuned with temperature, as observed in NiSi_2_ systems where the diffusion rate of Ni from the Ni source is enhanced with increasing temperature and in turn the silicide propagation length.^31^ In Figure 3 at 550 °C we can see the nonuniform and slower Ni diffusion rate, whereas at temperatures above 600 °C, faster Ni silicide growth progression was seen. With the progressing silicide propagation, the sample in Figure 5 was further annealed at 625 °C for 11 min. During this heating process (Figure 6a–h, Movie S4), the region indicated by the blue arrow on the left side of the NW (Figure 6a,b) moves across the growing silicide layer (Figure 6c,d). This moving region builds up on the right side of the NW in Figure 6e,f (red arrow), which provides a pathway with the lowest energy barrier. Subsequently, the region moves again to the center of the NW, gradually segregating from the silicide (green arrow) and accumulating in the Si segment in front of the silicide (Figure 6g). As the silicide grows, the region is pushed further into the Si segment within 11 min of annealing (Figure 6h). During this process, Ni traps in the Si NW continue to grow along the NW length, as can be seen in the HAADF-STEM image in Figure 4 at the bottom of the wire and also in the EDX maps in Figure S10 obtained after annealing.

In order to investigate the composition of the segregated region marked with the green arrow in Figure 6h, STEM-EDX elemental maps (Figure S11a–d) and spectra were acquired (Figure S11e), revealing the region composition as 88 at. % Si, 1 at. % Ni, and 11 at. % Sn. Interestingly these values differ from that of the composition of the starting NW before annealing with atomic fractions of 98 at. % Si and 2 at. % Sn (Figure S12). An explanation for this phenomenon is that due to silicide formation, the Sn present in the NW is redistributed. Because Sn is not incorporated in the silicide, it segregates from NiSi_2_ but at the NiSi_2_/Si interface (Figure S13). This observation is similar to the redistribution of dopants during silicide formation, which has been highlighted in previous studies of Ni–Si systems using atom-probe tomography (APT), *in situ* X-ray diffraction (XRD), and secondary-ion mass spectrometry (SIMS).^45−49^ It was suggested that due to the limited solubility of dopants in the silicide layer, the dopants migrate and accumulate at the silicide/Si interface, a behavior identified as interfacial segregation or the snowplow effect.^45−47^ Consistent with the literature, these results provide real-time TEM evidence of interfacial segregation at the silicide/Si interface.

### Kinked Si NWs

**Figure 7:**
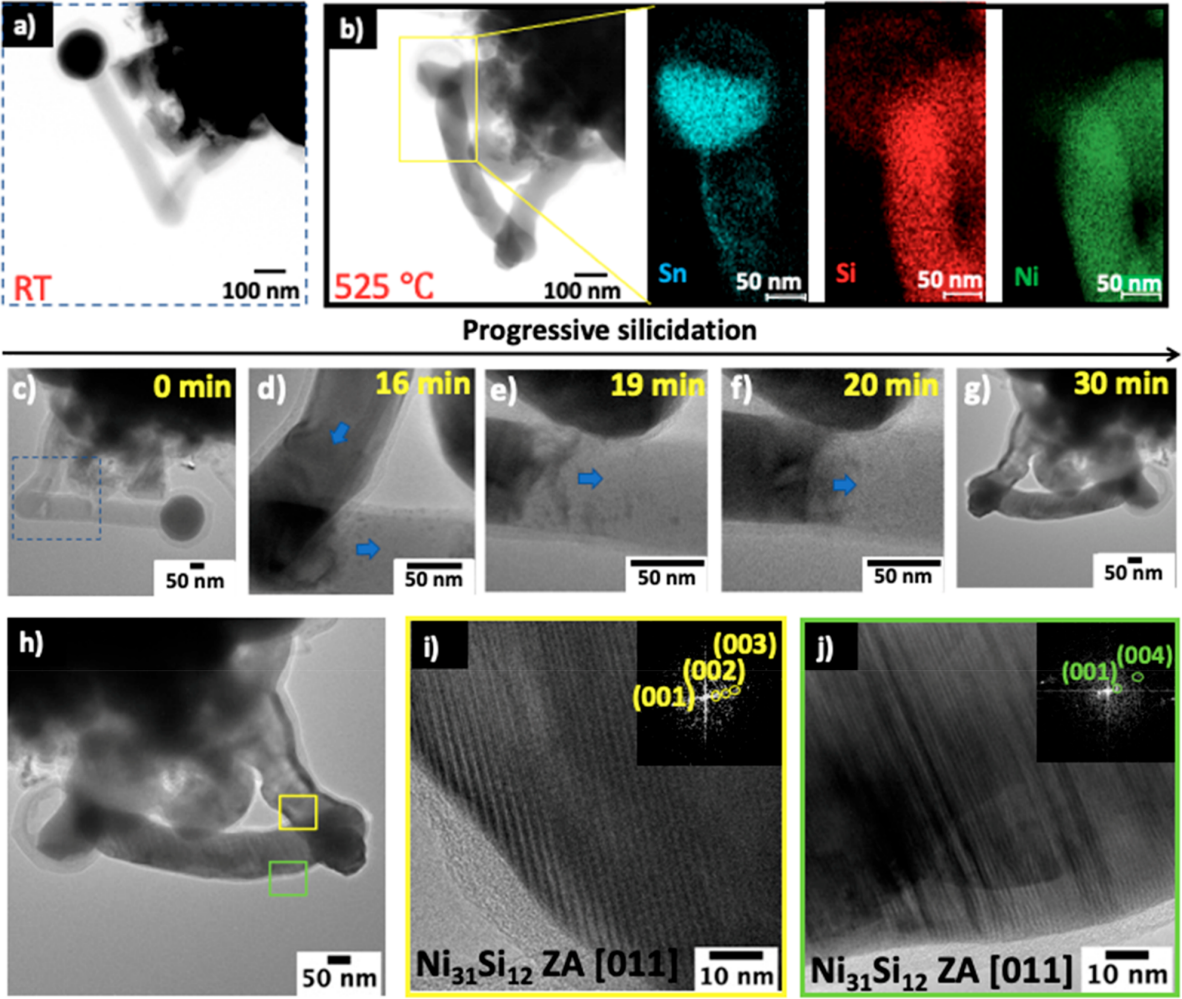
Low-magnification BF-STEM image of a kinked Si NW (a) before annealing and (b) after annealing at 525 °C with corresponding EDX elemental maps of Sn, Si, and Ni. (c–g) TEM snapshots of the progressive silicide formation during annealing. (h) TEM image of silicide NW. (i) HRTEM and FFT of the yellow box in h. (j) HRTEM and FFT of the green box in h.

In addition to straight Si NWs, the diffusion of Ni in kinked Si NWs was also studied. BF-STEM images of the sample before and after annealing at 525 °C for 30 min are shown in Figure 7a,b. The corresponding EDX compositional maps after annealing (Figure 7b) show that the Si NW (Figure 7a, Figure S14) has undergone a phase transition to a silicide NW. From the images captured during the annealing experiment (Figure 7c–g), Ni diffusion along the straight and kinked regions of the NW can be summarized in three stages: (i) Ni diffuses into the Si NW, and at a critical concentration, Ni silicide forms. (ii) Ni continues to diffuse and react with the Si at the silicide/Si interface. (iii) The silicide phase consumes the entire length of the NW. Ni_31_Si_12_ was identified as the silicide phase formed using the HRTEM images and corresponding FFT patterns in Figure 7h–j. This metal-rich phase can be attributed to the continuous supply of Ni atoms from Ni in contact with the sides and roots of the Si NW (Figure 7c–g).

Given the structure of the NW, it was expected that the Ni diffusion rate might be hindered at the kinked region of the NW or even lead to a nonuniform silicide growth. However, the observations in Figure 7 show a uniform growth behavior. The bending and overlapping of the NW provided contact and a continuous pathway for Ni diffusion. Another kinked Si NW observed in Figure S15a showed that during annealing Ni bypasses the kinked region highlighted with a red arrow in Figure S15b. In the other kinked regions with only a slight deviation from the straight path of the NW, Ni can diffuse around the kink and continue to form a silicide.

## Conclusion

In conclusion, *in situ* TEM heating experiments were performed to track Ni silicide formation in Si NWs, and our findings reveal the complex silicidation dynamics controlled by NW morphology, surface conditions, and crystallographic defects. The presence of kinks and planar defects in Si NW contributed to varying degrees of nonuniform silicide formation. Unlike defect-free straight Si NWs, Ni diffusion guided by planar defects including twin boundaries and stacking faults led to uneven silicide/Si interfaces. Such a silicidation process originates from the high strain field associated with the planar defect density in the NW. Our observations suggest that defect-driven silicidation has implications in terms of the quality of the silicide/Si interface as well as the overall material reliability for example in microelectronics applications, where the creation of highly abrupt contacts is crucial. The absence of impurities on the Si NW surface is also crucial for ensuring a uniform silicide formation as well as preventing silicide encroachment beyond the desired region of the Si NW during heterostructure formation.

Our results also showed interfacial segregation in a Ni silicide system when Sn is present in the NW. Sn segregation is expected to bring about modifications in the interface properties of the heterostructure, particularly the relationship between segregation and electrical conductivity. The concentration of Sn at the interface will likely dictate whether such properties are beneficial. Fine control of silicide phase, heterostructure location, and uniformity is required for advancing the development of next-generation nanodevices. The findings of this research provide evidence of interactions that control the growth kinetics of silicides in complex NW structures. Importantly, the insight gained from this research expands the existing knowledge on silicide formation in Si NWs and can also be extended to studies of metal diffusion in other 1D NWs.

## Experimental Methods

### NW Synthesis

Nickel NWs (PlasmaChem GmbH, Germany) with an average diameter of 200–300 nm and a length of 100–200 μm were used as hosts for the growth of Si NW branches via the solution–liquid–solid (SLS) mechanism. First, a mixture of 3 mg of Ni NW powder and 9 mL of squalane (99% purity, Sigma Aldrich Inc., USA) was sonicated for 30 min. Then, the Ni NWs/squalane solution was added into the long-neck Pyrex round-bottomed flask, which was connected to a condenser and sealed with a septum cap. The reaction setup was placed in a three-zone furnace and connected to a Schlenk line. All residual moisture was evacuated by heating the furnace to 125 °C under vacuum (<300 mTorr) for 30 min and then backfilled with Ar gas.

To initiate the seeded growth of branched Si NWs, Sn NPs purchased from Sigma Aldrich (>99% purity, *d* = 150 nm) were used as the growth catalyst. A suspension of Sn NPs in squalane (0.5 mg/1 mL) was injected through the top of the septum cap. The solution was then heated to 490 °C under a continuous Ar flow and reflux through a water condenser. During this period, the Sn seeds were formed on the Ni NW surfaces, and 0.75 mL of phenylsilane precursor (97%) was injected into the reaction flask. After 5 min, 0.02 mL of a (1:1) mixture of LiBH_4_ solution (2.0 M in THF, Aldrich) and squalane was injected to reduce surface oxides and facilitate NW growth. The decomposition of the precursor induced the nucleation and growth of the Si NWs from the sidewalls of the Ni NW templates with a total reaction time of 120 min. The flask was allowed to cool to room temperature under Ar flow and removed from the furnace.

### NW Washing Steps

NWs were extracted from the furnace by first sonicating the flask for 5 s to redisperse the NWs from the flask walls into the residual squalane solution followed by centrifuging the NW solution in a toluene/isopropanol (1:1) mixture at 5000 rpm for 10 min. This washing step was repeated two more times, and the precipitate was redispersed in ethanol.

### Sample Preparation for the *in Situ* TEM Experiment

The solution of NWs in ethanol was drop-cast onto a lacey carbon sheet (Cu grid). An FEI Helios G4 CX dual-beam microscope (FIB/SEM) with an Oxford X-maxN 50 EDX detector was used for imaging and sample transfer to a MEMs heater chip. A Thermo Scientific μHeater and DENSsolutions Wildfire/Lightning heating nanochips were used as the sample carrier for the *in situ* heating experiment. The MEMs chips were mounted on a specimen stub; the TEM grid was on a row-bar holder, and both were placed in the specimen chamber of the dual-beam microscope. Using the FIB system, a site-specific sample transfer routine was created, which involved (i) attaching the NW from the grid to the tip of a micromanipulator probe with carbon, (ii) transferring the probe to the selected window on the MEMs chip, (iii) lowering the probe until the NW came in contact with the MEMs chip, (iv) attaching the NW to the MEMs chip window with carbon, (v) cutting the NW free from the probe, and (vi) reinforcing the carbon weld between the NW ends and the MEMs chip. All steps were performed in SEM mode except step (v), which was performed with the ion beam but with a minimal dose (30 kV and 7.7 pA) and a total milling time of 20 s.

After sample transfer, the chip contacts were cleaned with deionized water. Then, the chip was placed in the built-in slot of a specialized *in situ* TEM heating holder (DENSsolutions Wildfire/Lightning TEM holder) and inserted into the TEM.

### *In Situ* Electron Microscopy Characterization

Control heating experiments were performed with a Thermo Scientific μHeater holder inside a FIB/SEM. Then TEM heating experiments using DENSsolutions Wildfire/Lighting systems were performed using an aberration-corrected monochromated FEI Titan Themis Cubed G2 60–300 kV TEM and an FEI Titan 80–300 kV FEG S/TEM. Both FEI microscopes were operated at 300 kV in STEM and TEM modes. High-angle annular dark-field scanning TEM (HAADF-STEM) images and high-resolution TEM of the NWs were recorded before and after heating experiments. STEM images were acquired with collection angles of 51–200 mrad, a 50 μm C2 aperture, a spot size of 9, a 17.9 mrad convergence angle, and a 91 mm camera length.

EDX STEM data for compositional analysis were also acquired using Super-X and Bruker XFlash 6-30 EDX detectors coupled with the microscopes. All temperature ramps were performed manually using the Digiheater software that accessed the heater control box. During annealing, the temperature was ramped up from 23 to 50 °C and then to 300 °C with an increment of 50 °C. Afterward, the temperature was increased by 25 or 50 °C and held for 2 to 5 min at each increment until phase transformation occurred. This phase transformation temperature was determined to be between 525 and 900 °C. The annealing temperatures were accompanied by uncertainties of ±0.004 °C.

All data acquired were processed with Thermo Fisher Scientific Velox software and Gatan Digital Micrograph software. STEM data were exported in TIFF 8, 16 bit, and video formats using Velox, and image frames from TEM data sets were aligned and summed using a digital micrograph in order to give single images with good signal-to-noise ratios. Further processing of TEM and STEM movies and images was performed using FIJI (ImageJ), an image analysis tool, to remove noise and enhance image contrast. Strain maps were extracted from HRTEM images using the software strain++, which measures the strain using the geometric phase analysis algorithm.

To determine the silicide phase formed, chemical analysis results in combination with FFT patterns and PXRD data were used. First, using STEM EDX, the atomic fraction % of elements within the region of interest was determined, with the consideration of a few percent uncertainty accounted for by rounding up to the nearest 2%. The observed atomic fraction % of Ni and Si in the silicide phase served as a guide for identifying an appropriate XRD file (PDF4+). Selected area FFT patterns obtained from HRTEM images were analyzed and indexed by comparing them with the identified reference XRD patterns. This stepwise approach allowed determination of the exact crystal structure and phase of each observed silicide.

## Supporting Information Available

Additional experimental data including heating profiles, SEM, TEM, HRTEM, STEM EDX elemental mapping, *in**situ* TEM images, strain maps, and a discussion of the Sn catalyst behavior during the heating experiments (PDF)
Movie S1: *in situ* STEM of the structural evolution of straight Si NW 1 annealed at 900 °C (AVI)
Movie S2: *in situ* STEM of uneven silicide growth behavior of straight Si NW 2 annealed at 550 °C (AVI)
Movie S3: *in situ* STEM of linear silicide growth behavior in straight Si NW 2 annealed at 600 °C (AVI)
Movie S4: *in situ* STEM of interfacial segregation in straight Si NW 2 annealed at 625 °C (AVI)

## Supplementary Material

nn4c01060_si_001.pdf

nn4c01060_si_002.avi

nn4c01060_si_003.avi

nn4c01060_si_004.avi

nn4c01060_si_005.avi
