# Supplementary material for: Real-Time TEM Observation of the Role of Defects on Nickel Silicide Propagation in Silicon Nanowires
Source: ACS Nano. 2024 Mar 21;18(14):10270–8. doi: 10.1021/acsnano.4c01060 (PMC11008354; doi:10.1021/acsnano.4c01060)
Supplement: Supplementary file 1 — nn4c01060_si_001.pdf [file nn4c01060_si_001.pdf]

Real time TEM observation of the role of defects on Nickel Silicide Propagation in Silicon Nanowires

Temilade Esther Adegoke [a], Raman Bekarevich [b], Hugh Geaney [a], Sergey Belochapkine [c], Ursel Bangert [d], Kevin M. Ryan *[a]

[a]*Department of Chemical Sciences and Bernal Institute, University of Limerick, Limerick, V94 T9PX Ireland.*

E-mail: kevin.m.ryan@ul.ie

[b]*Advanced Microscopy Laboratory, Centre for Research on Adaptive Nanostructures and Nanodevices (CRANN), Trinity College Dublin, D02 DA31 Ireland.*

[c]*Bernal Institute, University of Limerick, Limerick, V94 T9PX Ireland.*

[d]*Department of Physics and Bernal Institute, University of Limerick, Limerick, V94 T9PX Ireland.*

Supporting Information

Straight Si NW 1

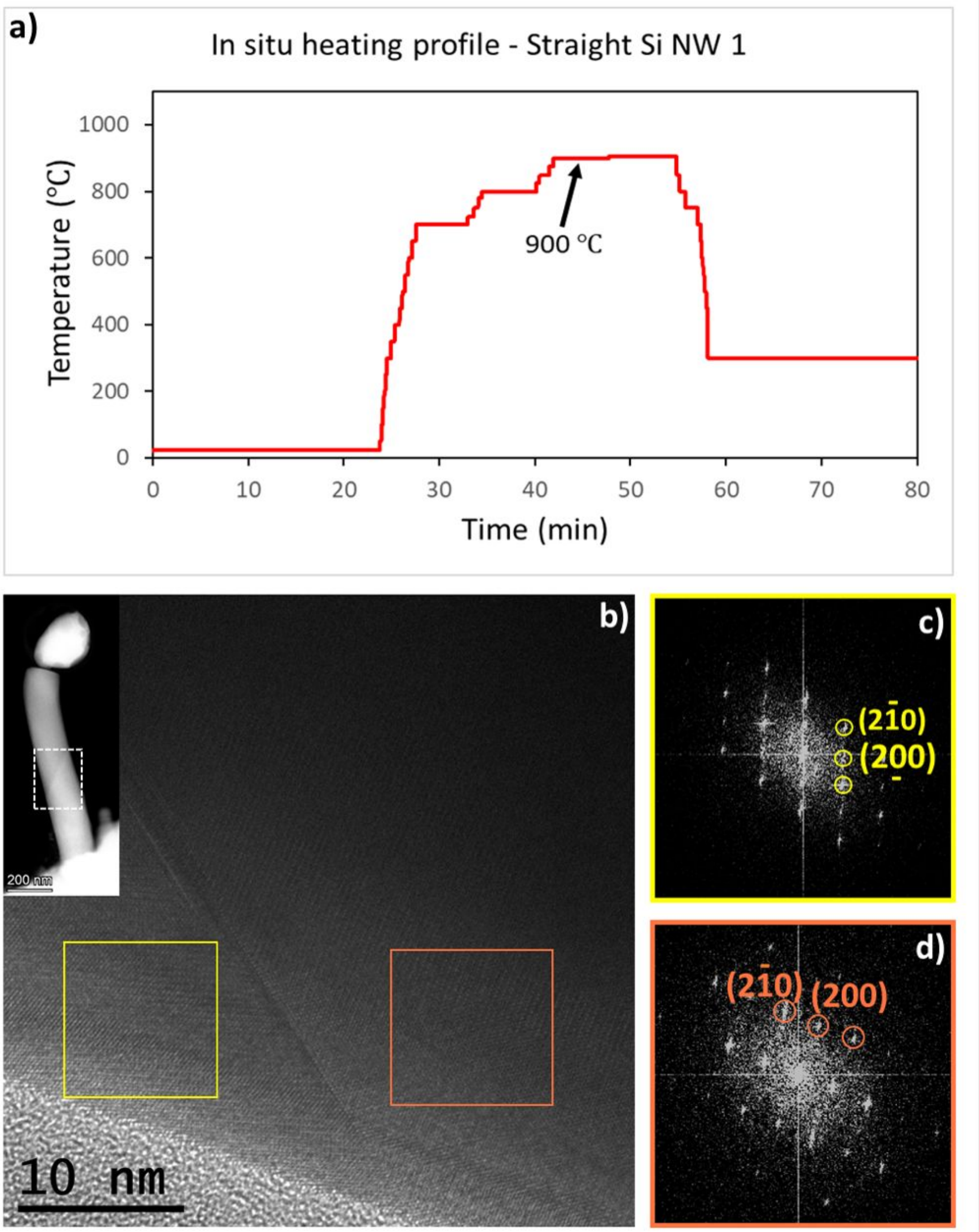

Figure S1. (a) Heating profile of <111> oriented straight Si NW 1 (b) HRTEM image taken from the [001] zone axis of the marked region in the inset NW image. (c) FFT pattern of the left region of the twin boundary in panel b (yellow box). (e) FFT pattern of the right region of the twin boundary in panel b (orange box).

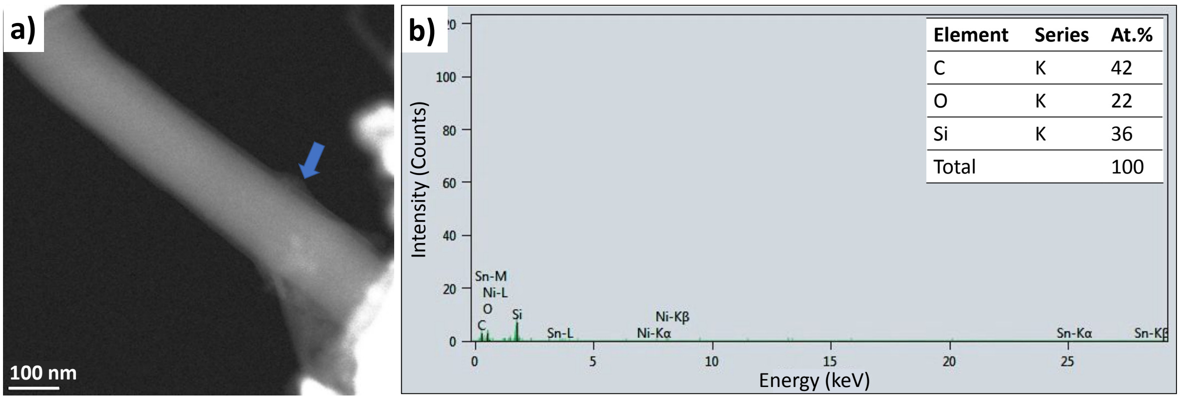

Figure S2. (a) HAADF STEM images of <111> oriented straight Si NW 1 before annealing with blue arrow highlighting the region of the NW with surface impurity. (b) STEM-EDX spectra and atomic fraction % of elements present in marked region in a.

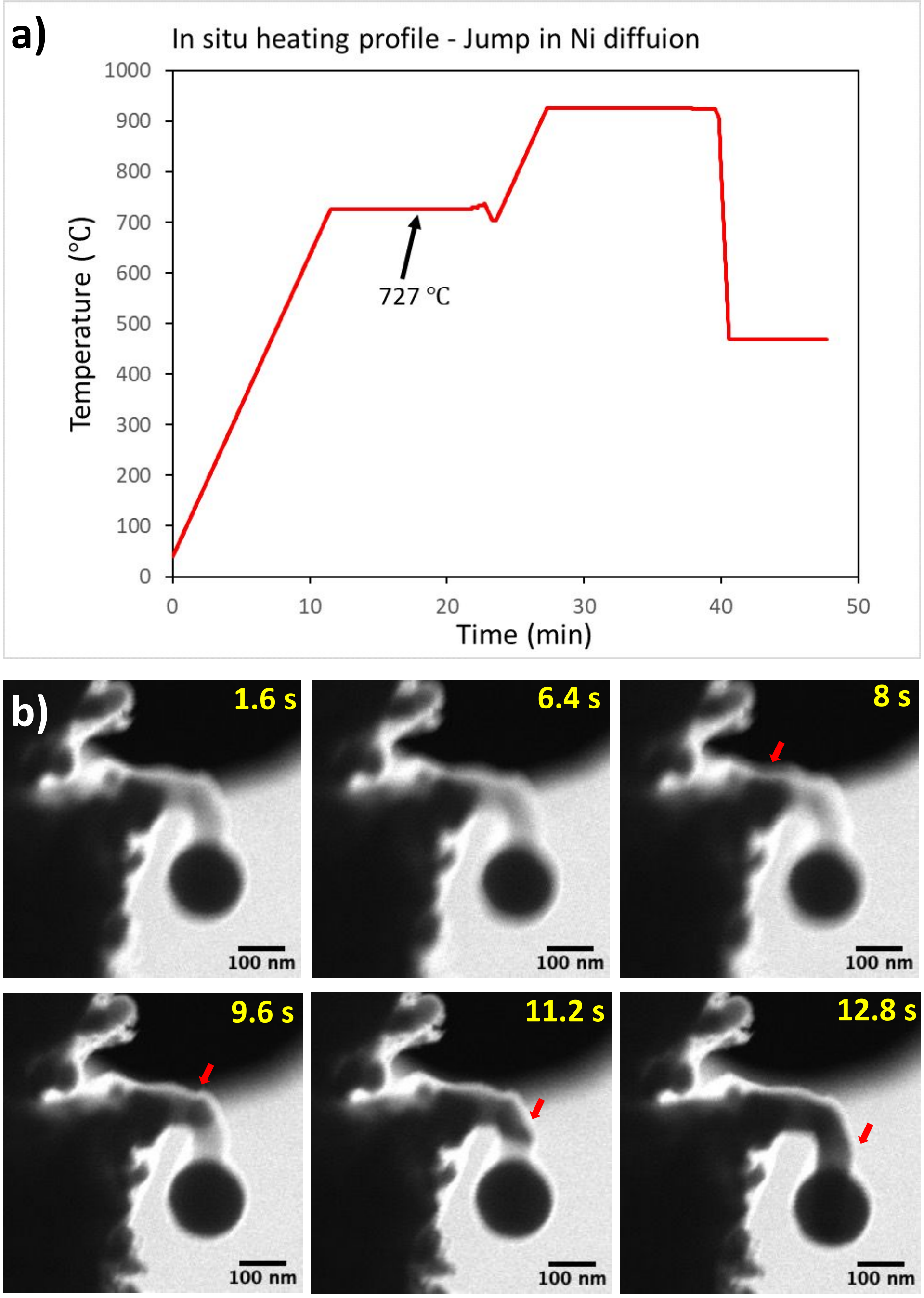

Figure S3. (a) Heating profile (b) Time-lapse bright-field STEM image of silicide formation in a Si NW captured using FIB-SEM. Red arrows highlight the jumps in Ni diffusion, with Ni consuming the entire length of the NW between 4.8 s to 12.8s.

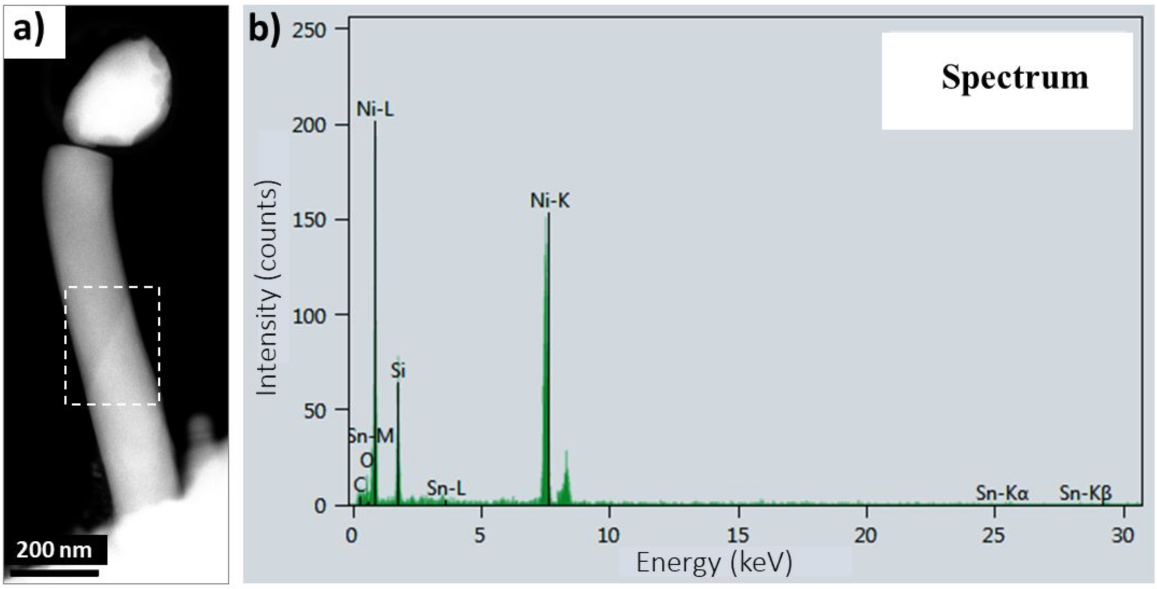

Figure S4. (a) HAADF- STEM image of <111> oriented straight Si NW 1. (b) STEM-EDX spectra of marked region in a.

Straight Si NW 2

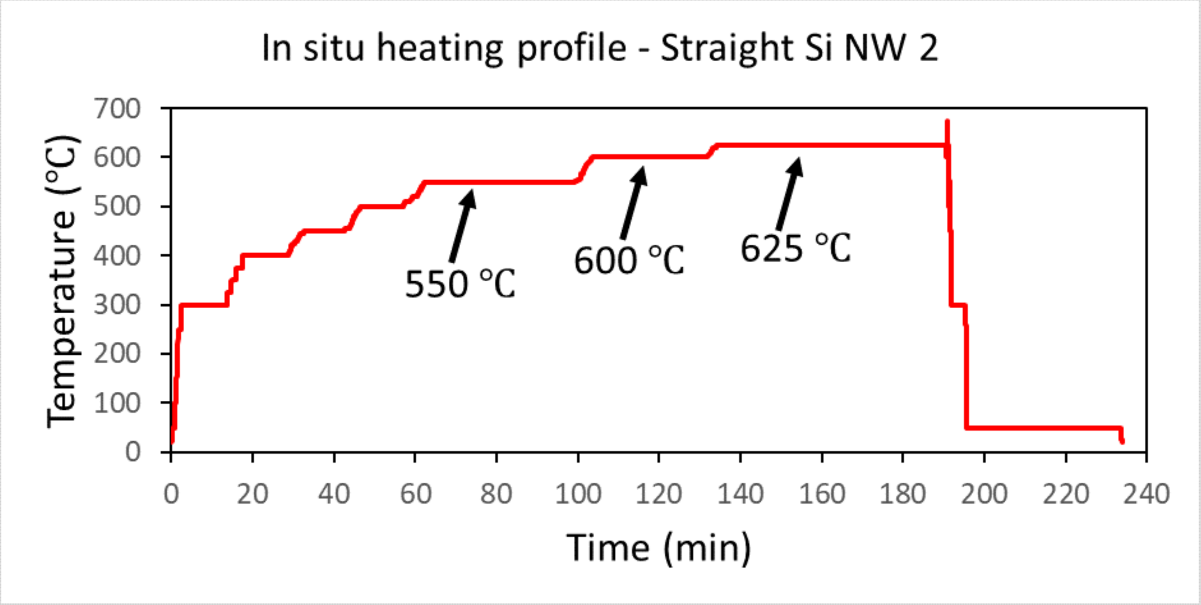

Figure S5. Heating profile of second <111> oriented straight Si NW 2 with phase transformation tracked at 550 °C, 600 °C and 625 °C respectively.

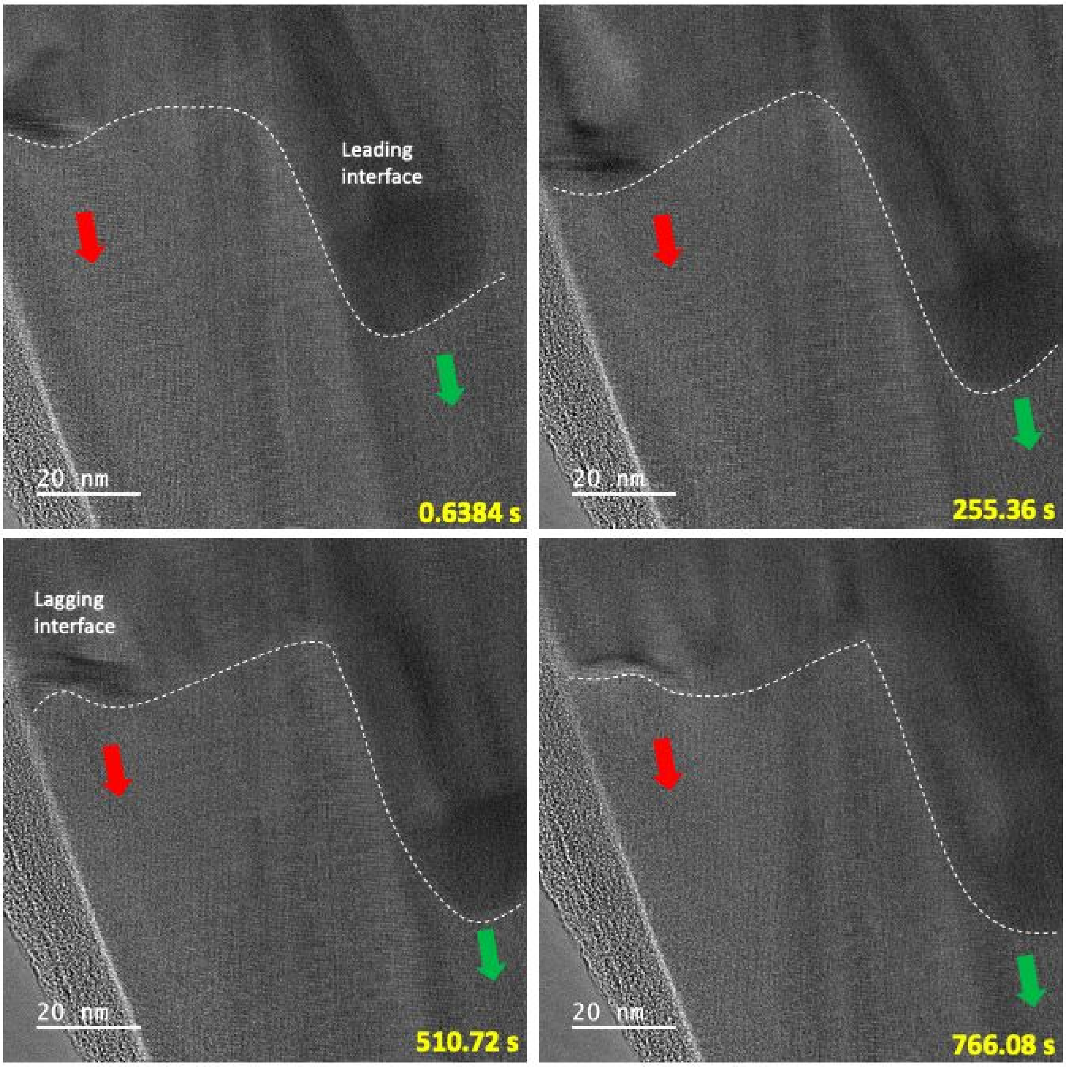

Figure S6. Time-lapse TEM image of an uneven silicide/Si interface in <111> oriented straight Si NW 2 annealed at 550 °C. Red and Green arrows represent the lagging and leading growth fronts from the edge to the centre of the NW. White dashed lines highlight the silicide/Si interface.

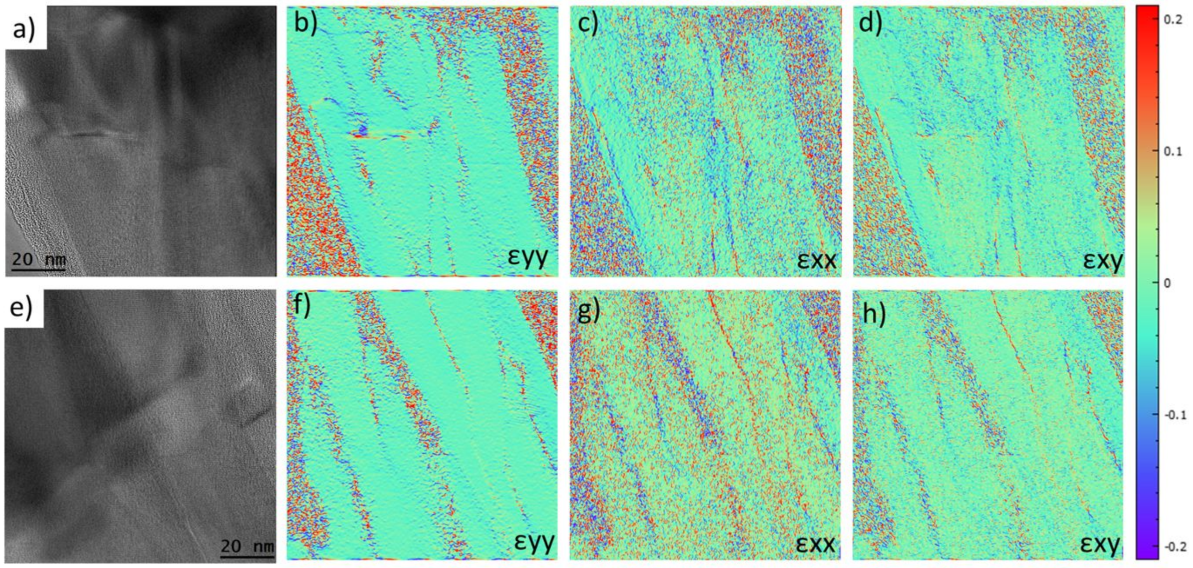

Figure S7. (a) HRTEM image of lagging interface. (b-d) εyy, εxx and εyx (shear) strain maps show the presence of strain along the silicide/Si interface. (e) HRTEM image of the leading interface (f-h) εyy, εxx and εyx (shear) strain maps. The colour variations from blue to red indicate the change in strain values from −0.2% to 0.2%, green represents zero strain. Regions surrounding the NW where lattice fringes are absent result in noise.

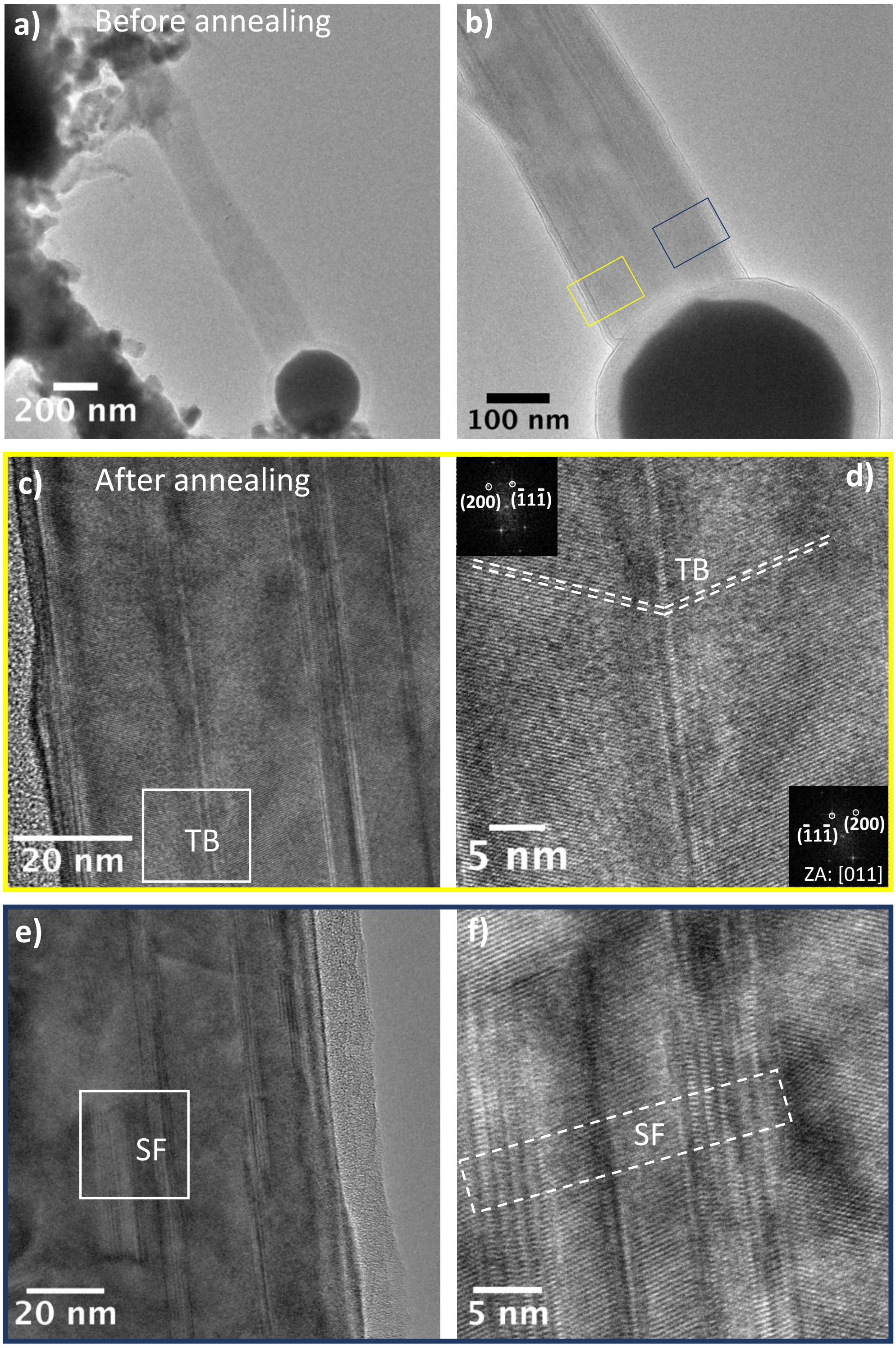

Figure S8. (a) Low magnification and (b) An over-focus TEM image of straight Si NW 2 before annealing. The defects are visible as dark lines along the NW (c) HRTEM image of the yellow box region in panel **b** showing the presence of multiple defects *i.e.*, twin boundary-TB (d) Atomic resolution image of the TB region in panel **c** and fast fourier transform image of both sides of the boundary. (e) HRTEM image of blue box region in panel **b**, showing the presence of multiple defects *i.e.,* stacking fault-SF (f) Atomic resolution image of SF region in panel **e**.

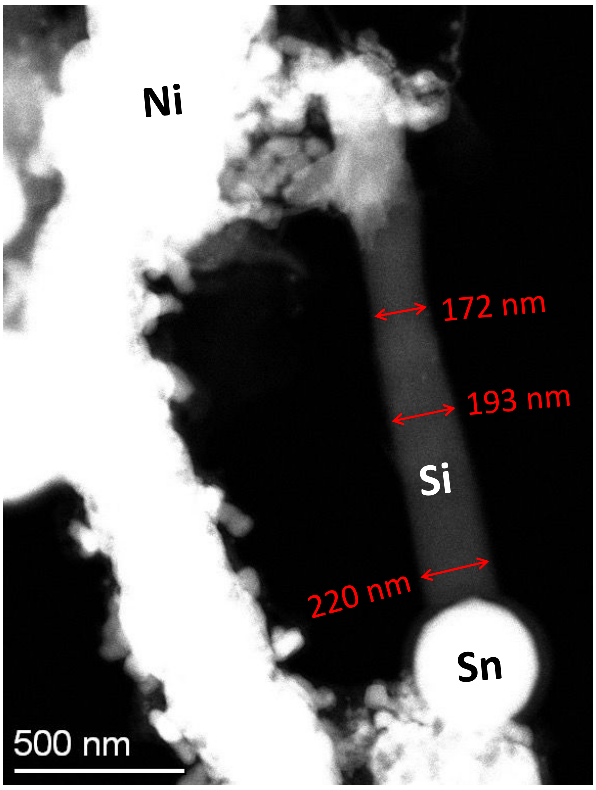

Figure S9. HAADF STEM image of <111> oriented straight Si NW before annealing showing a variation in diameter across the length of the Si NW.

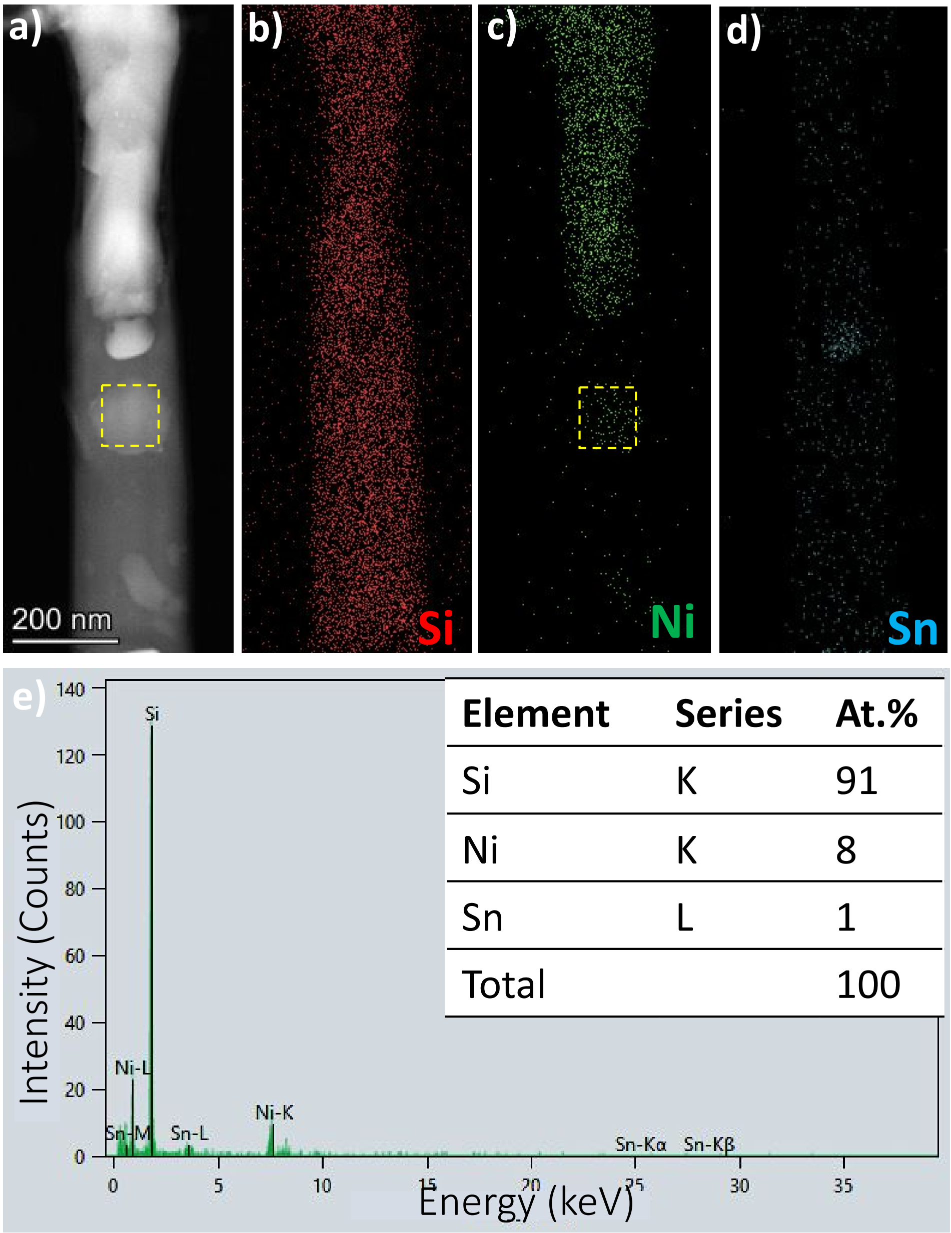

Figure S10. HAADF STEM image of <111> oriented straight Si NW 2 after annealing and corresponding EDX compositional maps of (b) Si red, (b) Ni green and (c) Sn blue. The yellow framed regions highlight the Ni traps. (d) EDX spectra of marked regions and the relative composition of Si, Ni and Sn in the NW.

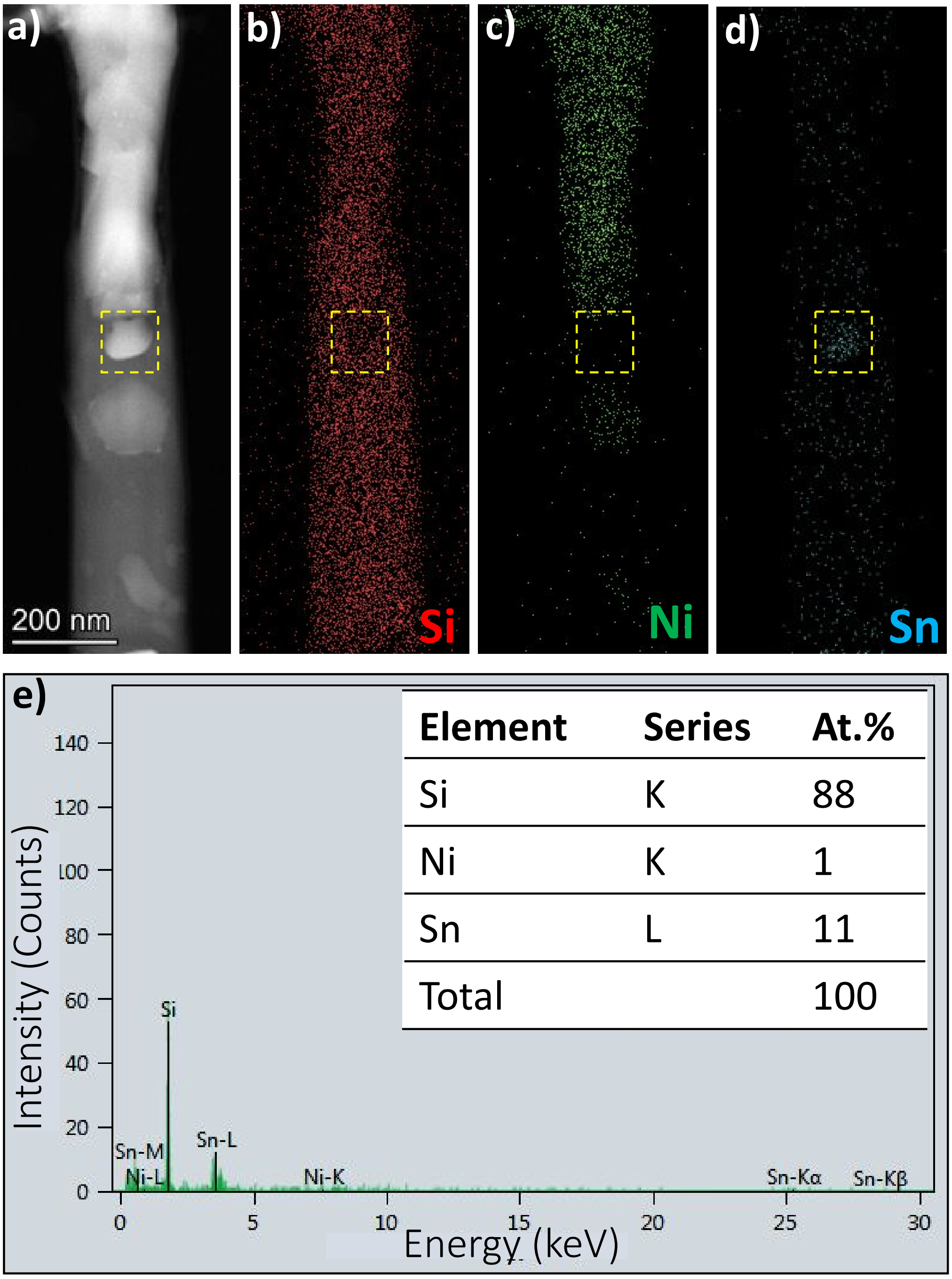

Figure S11. (a) HAADF STEM image of <111> oriented straight Si NW 2 after annealing and corresponding EDX compositional maps of (b) Si red, (c) Ni green and (d) Sn blue. Marked yellow regions highlight the phase segregation (e) EDX spectra of marked region and the relative composition of Si, Ni and Sn.

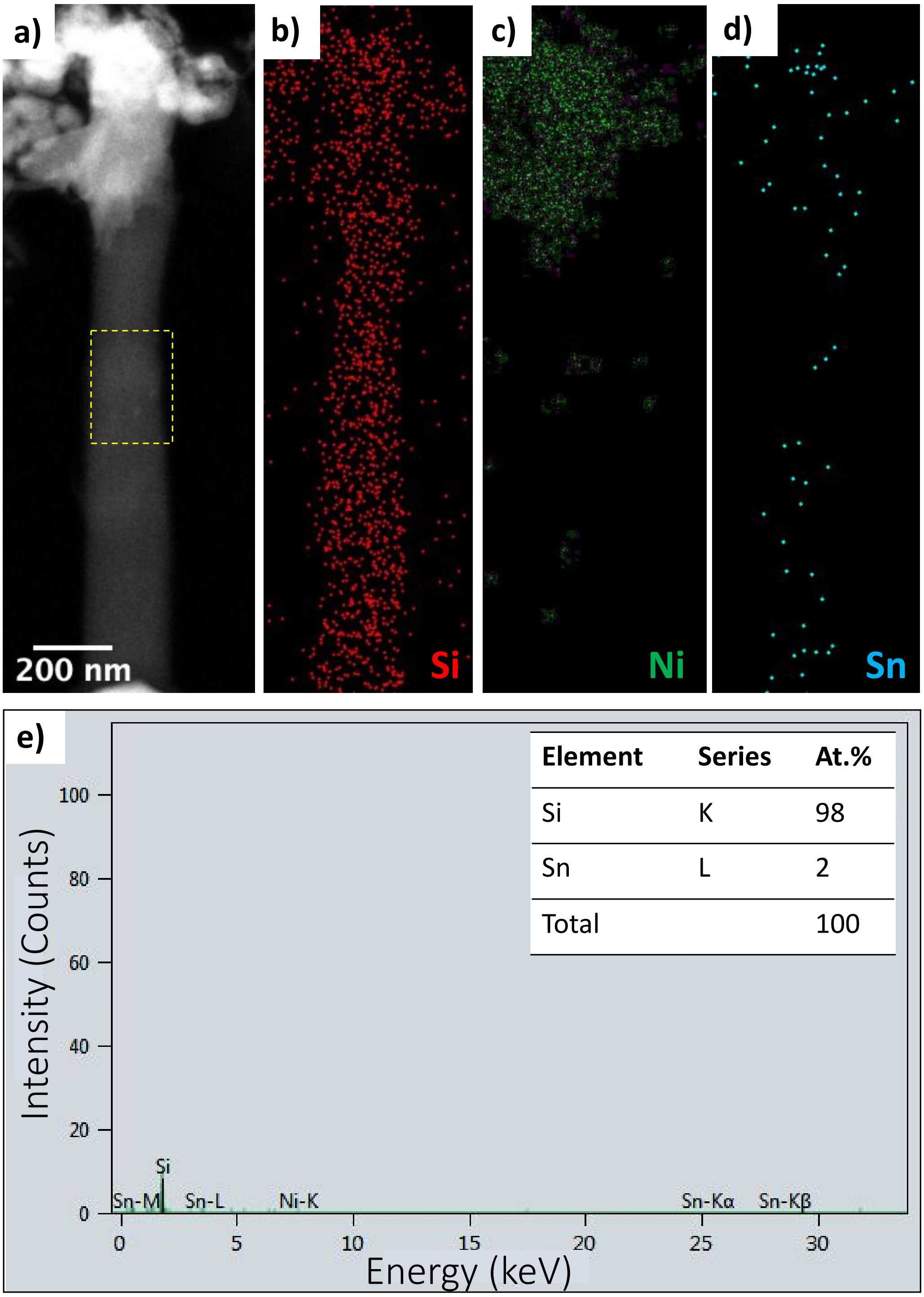

Figure S12. (a) HAADF STEM image of straight NW 2 before annealing and corresponding EDX compositional maps of (b) Si red, Ni green, Sn blue. (c) the relative composition of Si, Ni and Sn of the marked regions.

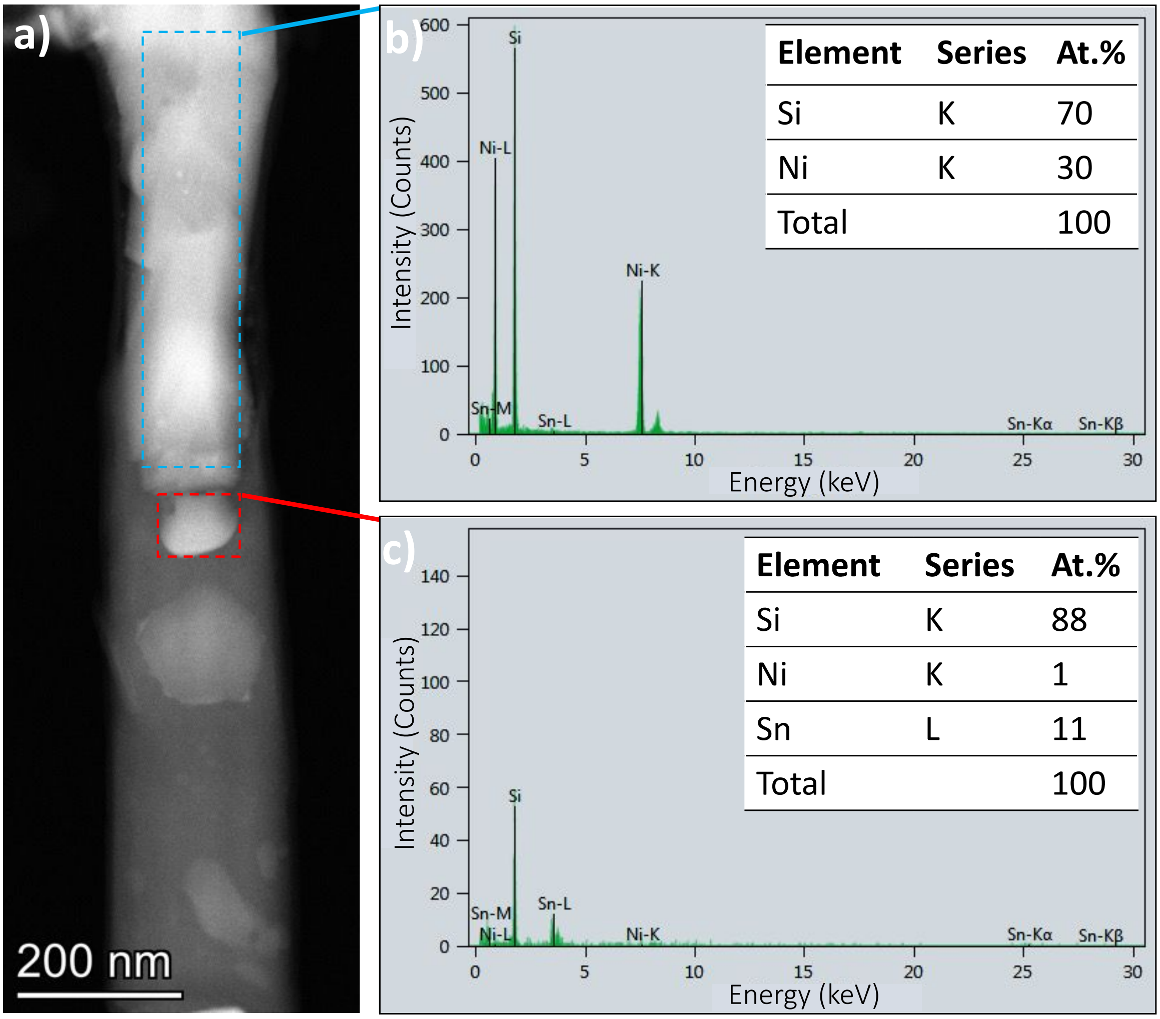

Figure S13. (a) HAADF STEM image of a NW after annealing with the corresponding spectra relative elemental compositions of marked regions in (b) silicide segment (c) segregated region.

Kinked Si NW

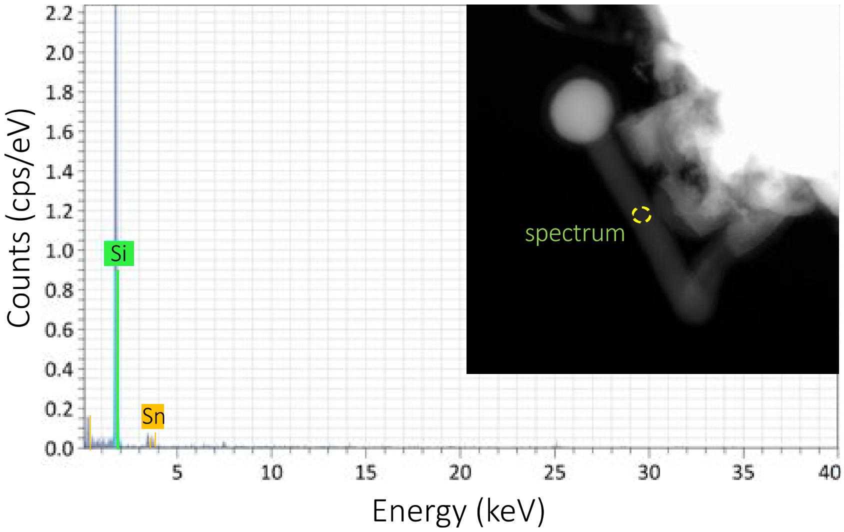

Figure S14. Low magnification Dark field STEM image and EDX spectra of a kinked Si NW 1 before annealing.

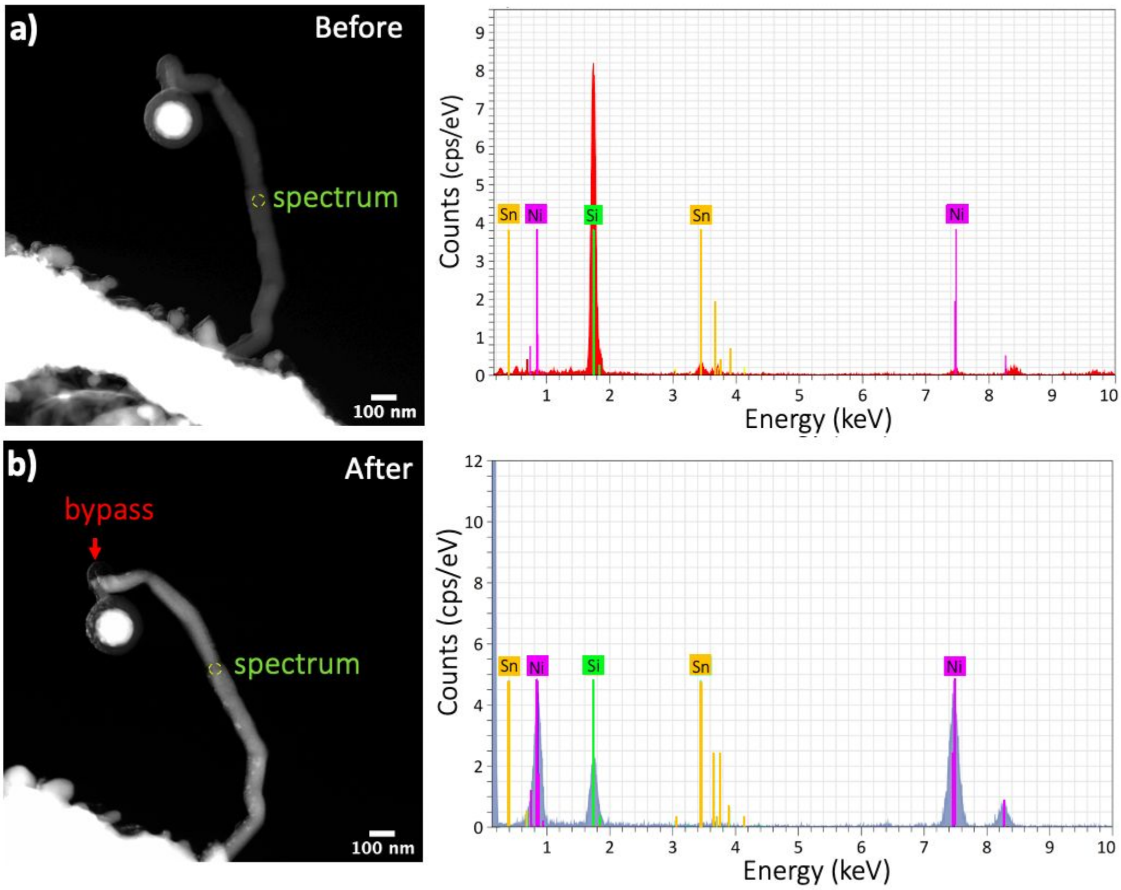

Figure S15. Low magnification HAADF-STEM images and EDX spectra of a Kinked Si NW 2 (a) before annealing (b) after annealing. The kinked region of the NW with no silicide formation is marked with a red arrow.

Sn Catalyst Behaviour

Throughout the annealing experiments, the Sn catalyst at the tip of the NWs has been observed to decompose gradually. Increasing temperature above 900 °C led to catalyst and NW decomposition. The representative TEM images of the morphological changes of the Sn catalyst are shown in Figure S16 at annealing temperatures ranging from room temperature (RT) to 600 °C, 700 °C, and 800 °C, A decrease in contrast at the edges of the Sn catalyst is first observed, followed by the formation of different sizes of Sn NPs. These NPs form preferentially at the edges and migrate to the surface of the Si NW. However, Sn NP migration was mostly limited to the regions exposed to the electron beam during the heating experiments. In some annealing experiments, the decomposition of the catalyst did not lead to Sn NP formation. It was found that at electron beam currents below 35pA did not promote the formation of Sn NPs observed in Figure S14, even at high temperatures of up to 900 °C. By changing experimental conditions such as the temperature, electron beam currents and time, the main parameters that influence the formation of Sn NPs were identified. We can conclude from the results so far that the rate of catalyst decomposition is dependent on both the annealing temperature and electron beam irradiation. While heat-induced decomposition has been observed in different studies[1], the effect of electron beam irradiation has also been shown to play a role in the decomposition and formation of a range of nanosized metal NPs.[2-5]

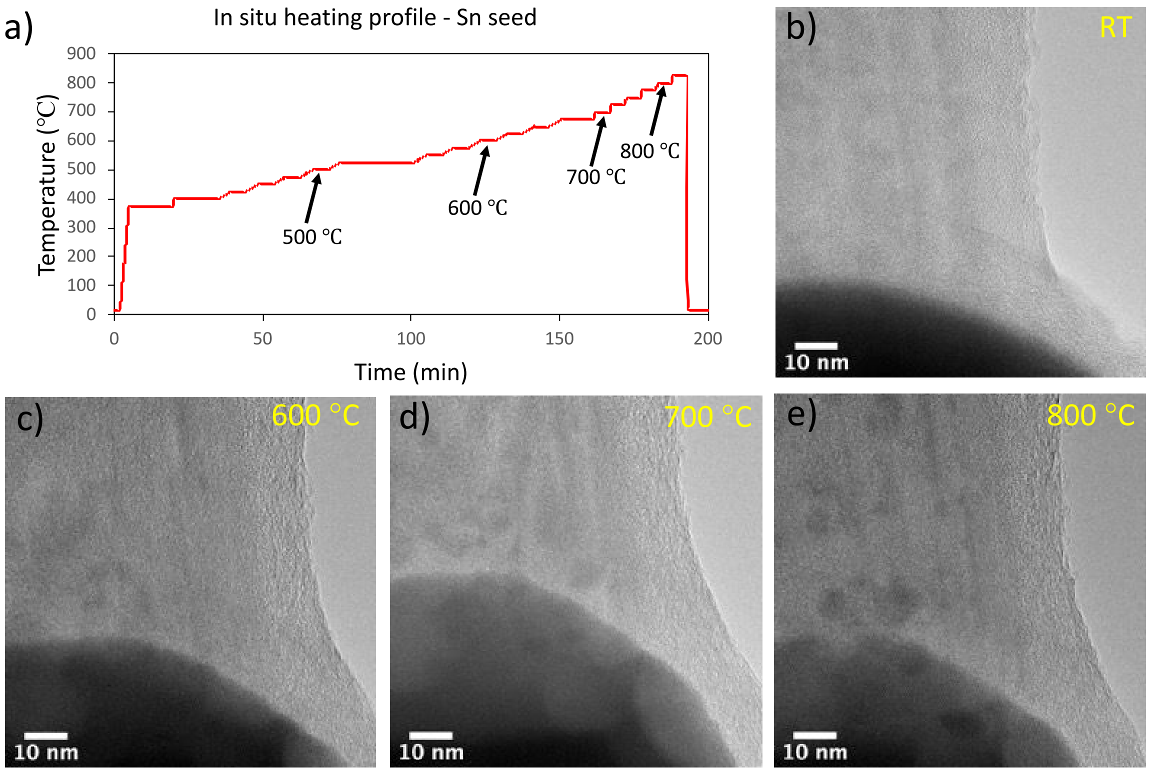

Figure S16. TEM snapshots of the structural evolution of the Sn catalyst as the annealing temperature is increased from room temperature to 800 °C.

References

1. Zhang, Z.; Liu, N.; Li, L.; Su, J.; Chen, P.-P.; Lu, W.; Gao, Y.; Zou, J. In Situ Tem Observation of Crystal Structure Transformation in Inas Nanowires on Atomic Scale. *Nano Letters* **2018,** *18*, 6597-6603, DOI: 10.1021/acs.nanolett.8b03231

2. Dang, Z.; Shamsi, J.; Palazon, F.; Imran, M.; Akkerman, Q. A.; Park, S.; Bertoni, G.; Prato, M.; Brescia, R.; Manna, L. In Situ Transmission Electron Microscopy Study of Electron Beam-Induced Transformations in Colloidal Cesium Lead Halide Perovskite Nanocrystals. *ACS Nano* **2017,** *11*, 2124-2132, DOI: 10.1021/acsnano.6b08324

3. Zhang, H.; Wang, W.; Xu, T.; Xu, F.; Sun, L. Phase Transformation at Controlled Locations in Nanowires by in Situ Electron Irradiation. *Nano Research* **2020,** *13*, 1912-1919, DOI: 10.1007/s12274-020-2711-2

4. da Silva Pereira, W.; Andrés, J.; Gracia, L.; San-Miguel, M. A.; da Silva, E. Z.; Longo, E.; Longo, V. M. Elucidating the Real-Time Ag Nanoparticle Growth on A-Ag 2 Wo 4 During Electron Beam Irradiation: Experimental Evidence and Theoretical Insights. *Physical chemistry chemical physics* **2015,** *17*, 5352-5359,

5. Sepulveda-Guzman, S.; Elizondo-Villarreal, N.; Ferrer, D.; Torres-Castro, A.; Gao, X.; Zhou, J. P.; Jose-Yacaman, M. In Situ Formation of Bismuth Nanoparticles through Electron-Beam Irradiation in a Transmission Electron Microscope. *Nanotechnology* **2007,** *18*, 335604, DOI: 10.1088/0957-4484/18/33/335604
